# Common mental disorders, productivity and presenteeism in nursing workers^*^

**DOI:** 10.1590/1980-220X-REEUSP-2022-0296en

**Published:** 2023-03-31

**Authors:** Raymara Melo de Sousa, Camila Maria Cenzi, Juliano Bortolini, Fábio de Souza Terra, Marília Duarte Valim

**Affiliations:** 1 Universidade Federal de Mato Grosso, Faculdade de Enfermagem, Cuiabá, MT, Brazil.; 2 Universidade Federal de Mato Grosso, Departamento de Estatística, Cuiabá, MT, Brazil.; 3 Universidade Federal de Alfenas, Escola de Enfermagem, Alfenas, MG, Brazil.

**Keywords:** Mental Disorders, Presenteeism, Efficiency, Organizational, Nursing, Occupational Health, Trastornos Mentales, Presentismo, Eficiencia Organizacional, Enfermería, Salud Laboral, Transtornos Mentais, Presenteísmo, Eficiência Organizacional, Enfermagem, Saúde do Trabalhador

## Abstract

**Objective:**

To investigate the association between the occurrence of common mental disorders with loss of productivity and presenteeism in nursing workers at a public health service.

**Method:**

This is a cross-sectional study, with 291 workers from Midwestern Brazil. Data collection carried out from October 2019 to January 2020, with instruments for sociodemographic characterization, labor and health conditions: Self Reporting Questionnaire-20, Standford Presenteeism Scale and Work Limitations Questionnaire. Data were analyzed using descriptive and inferential statistics, with Mann-Whitney tests and logistic regression, respecting a significance level of 5%.

**Results:**

The occurrence of common mental disorders was 4.27 times more likely to experience presenteeism, 10.17% of compromised overall productivity, and impairment of mental/interpersonal and production demands.

**Conclusion:**

The occurrence of common mental disorders was associated with presenteeism, with repercussions in loss of productivity of nursing workers.

## RESUMO

**Objetivo:**

Investigar a associação entre a ocorrência de transtornos mentais comuns com a perda de produtividade e o presenteísmo em trabalhadores de enfermagem de um serviço de saúde público.

**Método:**

Estudo transversal, com 291 trabalhadores da região Centro-oeste do Brasil. Coleta de dados realizada de outubro de 2019 a janeiro de 2020, com instrumentos para caracterização sociodemográfica, condições laborais e de saúde: *Self Reporting Questionnaire-20*, *Standford Presenteeism Scale* e *Work Limitations Questionnaire*. Os dados foram analisados por estatística descritiva e inferencial, com Testes de Mann-Whitney e regressão logística, respeitando o nível de significância de 5%.

**Resultados:**

A ocorrência de transtornos mentais comuns apresentou chances 4,27 maiores para vivenciar o presenteísmo, com 10,17% da produtividade geral comprometida e prejuízos para as demandas mental/interpessoal e de produção.

**Conclusão:**

A ocorrência de transtornos mentais comuns esteve associada ao presenteísmo, com repercussões em perda de produtividade dos trabalhadores de enfermagem.

## RESUMEN

**Objetivo:**

Investigar la asociación entre la ocurrencia de trastornos mentales comunes con pérdida de productividad y presentismo en trabajadores de enfermería de un servicio público de salud.

**Método:**

Estudio transversal, con 291 trabajadores de la región Centro-Oeste de Brasil. Recolección de datos realizada de octubre de 2019 a enero de 2020, con instrumentos de caracterización sociodemográfica, condiciones laborales y de salud: Self Reporting Questionnaire-20, Standford Presenteeism Scale y Work Limitations Questionnaire. Los datos fueron analizados mediante estadística descriptiva e inferencial, con pruebas de Mann-Whitney y regresión logística, respetando un nivel de significación del 5%.

**Resultados:**

La ocurrencia de trastornos mentales comunes fue 4,27 veces más probable de experimentar presentismo, con 10,17% de la productividad general comprometida y daños en las demandas mentales/interpersonales y de producción.

**Conclusión:**

La ocurrencia de trastornos mentales comunes se asoció al presentismo, con repercusiones en la pérdida de productividad de los trabajadores de enfermería.

## INTRODUCTION

Work is fundamental for biopsychosocial well-being. The current economic model has intensified the production process and resulted in work routines that require skills to deal with stressors that, when unbalanced, can trigger intense physical and mental exhaustion and, consequently, cause occupational illness, especially due to psychosocial disorders^(1,2)^. Neoliberal policy imposes a mode of production that reconfigures the organization of institutions and work techniques, such as making labor standards more flexible under market demand, streamlining activities, reducing costs, intensifying work and reducing wages^(3)^. In the nursing category, this fact increases the health system vulnerability and precarious working conditions^(4)^.

Working conditions are decisive for the health-disease relationship outcome^(5)^. With this, it is worth mentioning that nursing workers are the largest contingent in the field of health. Specifically in hospital work, they carry out continuous and uninterrupted monitoring and surveillance activities on users, articulating the work of other health professionals and ensuring care. In precarious work, the place occupied by these workers exposes them to greater suffering, as they are often unable to do everything they know is best for users^(6)^. Thus, achieving and maintaining nursing workers’ occupational health becomes a challenge, whose working conditions can lead to high levels of stress, trigger common mental disorders (CMD) and compromise performance^(1,7)^.

Recently, CMD represented the main cause of absence due to long-term incapacity for work in developed countries, with losses estimated at around US$ 16 trillion by 2030, worrying projections, since they are non-psychotic in nature, generally treatable and, in some cases, preventable^(8,9)^. They are represented by two main diagnostic categories: depression and anxiety, and also by symptoms such as sadness, neurasthenia, insomnia, fatigue, cognitive dysfunctions and somatic complaints. It should be noted that these disorders are often caused when the work process exceeds the adaptability of workers, enhancing feelings of dissatisfaction and little value, with significant losses in productivity^(10,11)^.

In Brazil, the occurrence of CMD occupies the third place in granting sick pay^(12)^. Presenteeism is increasingly representative, referring to workers who, even affected by some health condition/disease that may have repercussions on their productivity, remain physically present at work^(13)^.

In studies with health workers in which nursing was the most predominant category, presenteeism accounted for about 1.5 times more lost work time and compromised productivity four times more than absenteeism^(7,14)^. Among the various outcomes that the occurrence of CMD associated with presenteeism can trigger, the high chances of this worker suffering an accident at work, or even making mistakes that can endanger patient safety, stand out^(7)^.

In the literature, presenteeism is often considered a predictor of problems such as stress, burnout and exhaustion, as well as being associated with the occurrence of CMD and work commitment, whose costs caused by loss of productivity have been representing increasingly significant financial losses^(7,14)^. The association between the disease and the investigated phenomenon, in addition to being prevalent in the nursing area, are important early indicators for illness and future disability retirement^(13,15)^.

According to the World Health Organization Research Roadmap (WHO 2020), which encourages investigation of health workers’ mental health, based on the priority lines listed by the International Labor Organization (2022) for studies that assess the occupational risks to which nursing is exposed and the possible relationships between working conditions, productivity and quality of care^(16)^, and still according to the Ministry of Health research priorities^(17)^, the investigative question was formulated: is there an association between the occurrence of CMD with loss of productivity and presenteeism in nursing workers at a public health service?

It is believed that there is a shortage on this topic in the national context. Being able to act in the prevention of this phenomenon is positive for professionals, clients and organizations, with the possibility of reducing the risks of offering low-quality care as well as developing/intensifying occupational diseases. Therefore, this study sought to investigate the association between the occurrence of CMD with loss of productivity and presenteeism in nursing workers at a public health service.

## METHODS

### Study Design

This is a cross-sectional, analytical study, guided by the Strengthening the Reporting of Observational Studies in Epidemiology (STROBE) guidelines^(18)^, which contains a 22-item checklist with recommendations that alert the researcher about what should be included for a complete and accurate description of observational studies.

### Population

The population consisted of nursing assistants, nursing technicians and nurses from all segments of the unit where the research took place.

### Place

This study was carried out in a public hospital in midwestern Brazil, which includes outpatient services, hospitalization, therapeutic diagnostic support, emergencies, health surveillance. There are 37 surgical beds, 83 clinical, 25 pediatric, 40 adult Intensive Care Units (ICU), 5 pediatric ICU, 3 neonatal ICU, 7 for chronic patients and 3 for sanitary pulmonology.

### Sample Selection Criteria

At the time of data collection, there were 942 professionals registered at the institution, and 458 were nursing workers (127 nurses, 225 technicians and 106 assistants), in care and management positions. All who had at least six months of employment were included. All who were away for any reason were excluded.

### Data Collection

It took place from October 2019 to January 2020. The invitation and delivery of materials took place during working hours, with a limit of up to three attempts. Workers were invited individually by the researchers (nurses, with no ties to the data collection site/participants) and were informed about the objectives, relevance, voluntary compliance and confidentiality. Those who met the pre-established criteria, after signing the first copy of the Informed Consent Form (ICF), which remained with the researchers, received a sealed envelope containing four self-administered instruments and the second copy of the ICF. Material collection followed workers’ preference (deliver it in the same shift or in the next).

To characterize the sample, the semi-structured Sociodemographic, Work and Health Conditions Questionnaire (QSCTS – *Questionário Sociodemográfico, de Condições de Trabalho e Saúde*) was used, with 64 nominal and eight numeric variables^(10)^. It was constructed by nursing researchers based on studies that investigated factors related to presenteeism, validated in terms of face and content by judges with extensive experience in the areas of occupational health, mental health, nursing administration, in addition to a nurse and a nursing technician who had been working in care for about two years. The agreement index reached by expert judges was 0.90, and at the end of the process, 38 items were maintained, distributed in five categories.

The Self Reporting Questionnaire (SRQ-20) investigates the existence of non-psychotic morbidity through symptoms and emotional problems that occurred in the last 30 days. It consists of 20 dichotomous questions, which correspond, respectively, to the absence or presence of the symptom. The cut-off point represented the total of affirmative answers, where seven or more items indicate suspicion for CMD and in up to six questions, absence of CMD. The version validated in Brazil was used, with good specificity and sensitivity^(19)^. The achieved Cronbach’s alpha coefficient of SRQ-20 in this study was 0.86.

The Standford Presenteeism Scale (SPS-6) is indicated for studies that assess health conditions and productivity in the last 30 days. It is an ordinal scale with five alternatives analyzed by two dimensions with three items each. The score is calculated by summing: 6 to 18 - indicative of reduced performance; 19 to 30 - maintained work capacity. The validated version for Brazil was used, which showed good psychometric properties^(20)^.

The Work Limitations Questionnaire (WLQ) assesses how much a given health condition has impacted overall productivity and specific demands in the last 14 days. Consisting of 25 items grouped into four domains of limitation for work and for each one, the percentage of time commitment is calculated on a scale ranging from (0): no limitation to (100): limited all the time. After calculating the scores for each domain, the global rate of productivity loss is verified using a specific formula created by the authors of the original version. The validated version for Brazil was considered satisfactory and with good reliability^(21)^. Cronbach’s alpha coefficient reached in this research was 0.91.

### Data Analysis and Treatment

Of the 458 workers eligible for this study, 152 refused to participate, and 15 envelopes were disregarded because they contained incomplete instruments. Therefore, the rate of refusal and exclusion due to incompleteness totaled 36.4%, and the final sample had 291 workers. The data were double-checked into a database using Microsoft Excel^®^ and subsequently imported into the R software (R Core Team, 2021).

The QSCTS variables were described by frequencies, means, standard deviations (SD), minimums and maximums. Descriptive analyzes of the WLQ, SRQ-20 and SPS-6 variables were performed. The non-normal distribution was verified in the Shapiro Wilk test, and non-parametric tests were applied for comparative analyzes between groups. To assess the work capacity measured by the WLQ in relation to work and health aspects, comparative analyzes were carried out between the medians using the Mann-Whitney test. Regarding the scores related to the suspicion of CMD investigated by the SRQ-20, the associations with the QSCTS variables were investigated using two tests: logistic regression models and the Mann-Whitney test, which was also used in the comparative analyzes between the occurrence of the disorder and its impact on work capacity. Regarding the scores related to the occurrence of presenteeism by the SPS-6, associations were investigated with respect to the aspects investigated by the QSCTS and SRQ-20, through the Odds Ratios (OR) by simple logistic regression models, with significant associations if p-value ≤ 0.05 for the Wald test.

### Ethical Aspects

The recommendations of Resolution 466/12 of the Brazilian National Health Council were followed. The matrix project was approved in 2019 by the Research Ethics Committee (REC) of the *Universidade Federal do Mato Grosso*, Rondonópolis Campus (UFMT/CUR), under Opinion 3,217,476. The instruments were duly validated and provided, with prior authorization from those responsible for use in this research. There was no conflict of interest between the researchers and respondent collaborators.

## RESULTS

The sociodemographic and work characterization of the final sample of 291 nursing workers is described in Table 1, with a predominance of females, assistants/technicians, mean age of 39 years (SD = 9.5), single and with children.

**Table 1.** Distribution of sociodemographic and professional variables of nursing workers in a hospital and emergency room (n = 291) – Cuiabá, MT, Brazil, 2020.

Variables	n (%)
Sex	
Female	247 (84.88)
Male	44 (15.12)
Age	
Up to 39 years	164 (56.36)
40 years and older	127 (43.64)
Marital status	
Singles	130 (44.70)
Married	119 (40.90)
Widow or divorced	42 (14.40)
Children	
01 or more	202 (69.40)
None	89 (30.60)
Main family financial provider	
The respondent himself	227 (78.00)
Partner or parents	64 (22.00)
Contract regime	
CLT	174 (59.79)
Effective	106 (36.43)
Commissioned	11 (3.78)
Position	
Nursing assistant/technician	210 (72.16)
Nurse	81 (27.84)
Work sectors	
Adult/pediatric ICU and red/trauma room	123 (42.27)
Wards: medical, isolation, surgical	168 (57.73)
Shift and weekly workload	
Daytime: 12h/36h shifts or business hours	185 (63.57)
Night: 12/36 hour shifts	66 (22.68)
Night: 12/60 hour shifts	40 (13.75)
Other employment relationship	
No	218 (74.91)
Yes	73 (25.09)

As for health conditions, 17 (5.84%) reported having a diagnosis of generalized anxiety disorder (GAD), while 18 (6.19%) reported depression, 37 (12.71%) reported having anxiety associated with depression, which characterizes them with mixed anxiety and depressive disorder, 20 (6.87%), migraine, and 21 (7.22%), gastritis. Using anxiolytics, antidepressants or psychoactive drugs was reported by 61 (20.96%) workers. Of the sample, 34 (11.68%) self-declared smokers for about 11.3 years (SD = 7.42; min = 1; max = 30) and consumed a mean of 9.58 cigarettes/day (SD = 8.33; min = 2; max = 20). Furthermore, 83 (28.52%) reported drinking alcohol for about 9.6 years (SD = 7.19; min = 1; max = 40) and consuming on average 1.57 times a week (SD = 0.83; min = 1; max=7).

Therefore, 43 (14.78%) participants reported having a history of sick leave in the last year and the sick leave period lasted a mean of 39 days (SD = 50.8; min = 1; max = 210). Furthermore, 176 (60.48%) workers reported having a leisure routine and the frequency with which they practiced had a mean of 1.6 times a week (SD = 0.49; min = 1; max = 2).

The prevalence of presenteeism was represented by 111 (38.14%) workers who reported experiencing the phenomenon in the last 30 days. Of this total, 62 (55.86%) maintained their work capacity even with some health problem, while 49 (44.14%) carried out their activities with reduced work performance during the period in which they were affected by health problems.

The prevalence of symptoms suggestive of CMD was identified in 68 (23.37%) workers. The assessment of possible impacts on work capacity showed a global rate of loss of productivity of 10.13%, with a WLQ score index = 0.08. When presenting the impacts for each commitment domain, skills for specific tasks were limited in 40.27%, for physical demand, 30.78%, for time management, 30.38%, for production demand, and 29.55%, for mental/interpersonal.

Comparative analyzes between sociodemographic, work and health aspects in relation to the occurrence of CMD and presenteeism are presented in Table 2, with reference to the affirmative answers in relation to the negative ones. Therefore, the variables that were associated with the condition and the phenomenon refer to workers with children, diagnosed with depression, migraine and mixed anxiety and depressive disorder, in addition to those with a history of sick leave in the last year, which was not observed when there was a leisure routine.

**Table 2.** Associations between the variables Sociodemographic, Working Conditions and Health Questionnaire (QSCTS) with common mental disorders and presenteeism in nursing workers (n = 291) – Cuiabá, MT, Brazil, 2020.

Variables (Yes x No)	OR	(95%) CI	p-value*
**Common mental disorders**			
Children	2.79	1.43 – 5.89	<0.01
Depression	4.63	1.75 – 12.65	<0.01
Migraine	7.29	2.85 – 20.23	<0.01
Generalized anxiety disorder	9.34	3.32 – 30.38	<0.01
Mixed anxiety and depressive disorder	5.78	2.82 – 12.09	<0.01
Gastritis	6.35	2.55 – 16.77	<0.01
Medical leave in the last year	4.60	2.34 – 9.13	<0.01
Night shifts 12/36h	2.35	1.28 – 4.27	0.01
Night shifts 12/60h	0.94	0.40 – 2.03	0.89
Night shifts 12/36h and 12/60h	1.94	1.11 – 3.37	0.02
Double shift	2.53	1.41 – 4.54	<0.01
Smokers	3.04	1.43 – 6.37	<0.01
Alcoholics	2.76	1.56 – 4.88	<0.01
Leisure routine	0.93	0.53 – 1.62	0.80
**Presenteeism**			
Children	3.08	1.76 – 5.61	<0.01
Continuous psychotropic drug use	1.78	1.00 – 3.16	0.05
Depression	3.52	1.76 – 5.61	0.01
Migraine	2.61	1.04 – 6.86	0.04
Mixed anxiety and depressive disorder	2.72	1.35 – 5.60	0.01
Leisure routine	1.07	0.66 – 1.74	0.78
Medical leave in the last year	3.71	1.90 – 7.49	<0.01

*Logistic regression model – Odds Ratio.

Table 3 shows the comparative analyzes between work and health aspects in relation to the occurrence of CMD and loss of productivity, highlighting the existence of a diagnosis of gastritis, a condition that was associated with the condition and showed commitment to tasks related to mental demand/interpersonal.

**Table 3.** Comparative analysis between the variables Sociodemographic, Working Conditions and Health Questionnaire (QSCTS) with common mental disorders and nursing workers’ productivity (n = 291). Cuiabá, MT, Brazil, 2020.

Productivity – CMD	Yes		No		p-value*
QSCTS	Median	(SD)	Median	(SD)	
**Work Limitations Questionnaire**					
General productivity index					
CLT	8.56	(6.80)	4.90	(6.15)	<0.01
ICU and emergency departments	8.17	(6.45)	6.53	(6.82)	0.04
Leisure routine	5.39	(6.45)	8.27	(6.78)	0.03
Time management					
CLT	30.00	(30.6)	15.00	(25.51)	0.01
Physical demand					
Night shift	37.50	(28.68)	30.00	(30.69)	0.05
ICU and emergency department	41.67	(25.77)	30.00	(31.47)	
Mental/interpersonal demand					
CLT	30.56	(28.58)	9.38	(23.57)	<0.01
Gastritis diagnosis	30.56	(22.62)	19.44	(27.81)	0.03
Production demand					
CLT	35.00	(30.97)	10.00	(28.36)	<0.01
**Common mental disorders**					
Children	3.00	(4.18)	1.00	(3.3)	<0.01
Second link	5.00	(4.63)	2.00	(3.62)	<0.01
Smoking	5.00	(5.45)	3.00	(3.68)	0.01
Alcohol use	5.00	(4.84)	2.00	(3.4)	<0.01
GAD diagnosis	9.00	(5.67)	3.00	(3.64)	<0.01
Depression diagnosis	7.50	(5.48)	3.00	(3.74)	<0.01
Migraine diagnosis	9.00	(5.44)	3.00	(3.66)	<0.01
Gastritis diagnosis	8.00	(4.62)	2.00	(3.65)	<0.01
Psychotropic drug use	5.00	(4.52)	2.00	(3.75)	<0.01
Health leave in the last year	5.50	(4.66)	3.00	(3.91)	0.03

*Mann-Whitney test.

Associations between CMD, presenteeism and productivity were investigated using logistic regression and the Mann-Whitney test. The results showed that workers with CMD were four times more likely to experience presenteeism compared to those classified without the condition ((p-value < 0.01) and (OR = 4.27; CI = 2.43–7.67)). The medians of the groups that investigated the phenomenon were compared and showed an association between the occurrence of CMD (p-value < 0.01) and presentee workers (md = 5; SD = 4.40), when compared to non-presentee workers (md = 2; SD = 3.29). In order to analyze the impact of injury on productivity, the medians of the groups of workers with and without suspicion of CMD were compared and the results showed impacts not only on general productivity, but on the performance of specific tasks, as shown in Table 4.

**Table 4.** Comparative analysis between the Work Limitations Questionnaire (WLQ) and Self-Reporting Questionnaire (SRQ-20) variables of nursing workers (n = 291) – Cuiabá, MT, Brazil, 2020.

Variables	General index		Time management		Physical demand		Interpersonal mental demand		Production demand	
	Mean (SD)^†^ Median	p-value	Mean (SD)^†^ Median	p-value	Mean (SD)^†^ Median	p-value	Mean (SD)^†^ Median	p-value	Mean (SD)^†^ Median	p-value
**SRQ-20**										
With CMD	10.13 (5.60) 8.59	0.02	34.00 (29.38) 30.00	0.29	45.75 (28.06) 41.67	0.06	34.70 (24.59) 30.56	0.01	34.67 (25.79) 31.25	0.02
Without CMD	8.58 (6.96) 6.28		29.79 (28.97) 22.50		38.59 (29.78) 35.00		27.98 (28.24) 16.67		29.08 (31.86) 15.00	

*n = sample number; ^†^SD = standard deviation.

## DISCUSSION

Workers with CMD were four times more likely to experience presenteeism, while being presenteeistic was also associated with the occurrence of CMD. It was found that the existence of CMD compromised general productivity and skills related to mental/interpersonal and production demands. Such findings were based on hypotheses in which CMD are predictors of loss of productivity, as they are associated with presenteeism^(12,15,22–24)^. The results of a study carried out with 1,218 nursing workers in Brazil suggest that work impaired by presenteeism mediated the association of high psychological demands and low social support with CMD. High psychological demands associated with reduced ability to concentrate at work due to a health problem were related to higher CMD scores^(15)^.

Sociodemographic aspects found were similar to those of a study carried out in Brazil on the importance of nursing in the Unified Health System, noting that the profession is represented mostly by females, aged up to 49 years, with children, single and mostly distributed in the categories of assistant/technicians^(25)^. It was observed that the variable “having children” increased the chances for the occurrence of CMD and presenteeism. Motherhood can be considered a risk factor for both because it causes family-work conflicts and because it requires extreme responsibility and skill in managing tasks considered (but little recognized) as second and third shifts^(5)^.

As for working and health conditions, it is noteworthy that double employment, night shift (12/36h) and consumption of alcohol and tobacco were associated with CMD. However, working in shifts can result in an imbalance in the circadian cycle, in the production of melatonin and cause sleep disorders, this is prevalent in nursing, the repercussions of which are worrying, since in a recent study 76.5% of the sample had poor sleep quality problems, while 41.8% had the aforementioned disorders^(26,27)^.

Previous studies showed that night work increases vulnerability to the occurrence of CMD^(7,26)^, and in nursing, represented greater wear, damage to sleep, rest and general well-being, conditions that can lead to physical and psychological impairment, corroborating with the findings in this study^(7,26,28)^. However, the chances were greater when CMD symptoms were presented by workers working 40 hours of the referred shift, i.e., night watchful shifts on alternate days performing equally demanding routines, in line with recent scientific evidence^(11,28,29)^.

Double employment is often motivated by the need to supplement the family income. They are routines highly susceptible to the occurrence of CMD because they result in long journeys represented by two contexts: keeping workers in a state of alertness and full attention increases the production of stress hormones for prolonged periods and the consequent lack of time for rest, sleep, physical and leisure activities compromise fundamental mechanisms for full recovery^(28)^.

In this study, work at night and in the ICU and emergency room demonstrated to limit physical capacity, with losses in productivity. CLT (registered with contract) workers showed loss of overall productivity. For mental/interpersonal and production demands, results that were in line with a study carried out with nurses from nine ICUs in the Brazilian Northeast, which found that the most frequent employment relationship was salaried and found a positive association between high workload and minor psychological disorders^(29)^.

Sectors such as ICU and emergency are characterized as stressful environments, with exhausting routines, intensive care, living with traumatic situations, pain and human suffering, which requires extreme emotional control^(1,2)^. It represents a context with high demands susceptible to musculoskeletal problems, which justifies the physical limitation presented in this study^(14,28)^. Therefore, when there is an excessive and prolonged consumption of physical and emotional resources that have not yet been fully recovered, there is an accumulation of fatigue and stress that can potentiate psychosomatic symptoms, impact productivity and result in burnout, apathy, illness or work incapacity^(7,14)^.

The instability in the employment relationship caused a feeling of insecurity that led to a permissiveness where sometimes, workers undergo strenuous conditions and extreme resistance to being absent even when they are sick, becoming competitive and conflicting environments, in an incessant search for high productivity motivated by fear of unemployment, which is why they represent worrying impacts on mental health^(1,2,11)^. This unhealthy work context encourages the results obtained by the damage to cognitive, interpersonal and organizational tasks, skills directly affected in the process of mental illness and it should be inferred that this situation has worsened even more with the COVID-19 pandemic. An important study showed that presentist workers affected by CMD reported being afraid to expose their health condition for fear of losing their jobs and that they did not feel comfortable even to take time off from work^(24)^.

Moreover, the intensity of work added to the vulnerability of new forms of contract and loss of rights increases and reinforces the social precariousness of work^(30)^. Research carried out in hospitals in Bahia found that nursing is subject to precarious working conditions and the intensity of work produced by the accumulation of employment relationships predisposes these workers to physical, mental and psychological fatigue, which can contribute to the occurrence of errors in care^(6)^. By rescuing the context of the neoliberal policy progressiveness instead of improvements in working conditions, nursing faces intense precariousness, which can impact the mental health of these workers, resulting in psychic suffering, expressed by feelings of sadness, fear, irritability and anguish^(4)^. It is noteworthy that during the COVID-19 pandemic, this precariousness of nursing work and unfavorable conditions in the work environment were more present.

Among the identified health variables, workers diagnosed with GAD, depression, migraine, history of sick leave, continuous use of psychotropic drugs and the lack of a leisure routine were associated with CMD and presenteeism, while problems with gastritis increased the chances for the occurrence of CMD and limited the capacity for demands that require cognitive skills and interpersonal relationships. It should be noted that depression is classified by the WHO as the disease that most contributed to global illness^(5)^. However, even though it is an internationally known relationship and that CMD represent a 33% greater chance of absenteeism, depression is often more associated with presenteeism^(7,22)^, which also occurred in this research.

In the international literature, it was observed that the costs with the loss of productivity caused by depression represented losses, and the most affected countries were the USA and Brazil, whose monetary loss was 5 to 10 times greater than absenteeism^(24)^. In Australia, the problem annually represents about US$ 10 to 15 billion in lost productivity^(26)^, with nursing being a prevalent category in the occurrence of these disorders^(7,13)^.

Gastrointestinal disorders are known among the psychosomatic symptoms of stress and are correlated with exhaustion, which would justify the results obtained having mainly psychic impacts and the hypothesis that psychosocial stressors may also contribute to the illness in question^(15,16)^. When there is a cognitive decline, the work performed is hampered by the extreme difficulty in processing, concentrating, remembering important principles for care, being organized, performing all the tasks provided with quality, predict future situations and thus result in risks to the patient, contamination of infectious diseases, occupational accidents, among other harmful outcomes^(14)^.

Nursing is recognized for tending to ignore symptoms of illness such as exhaustion, stress, fatigue, often being confused with tiredness from naturally exhausting routines. They are self-critical professionals who have a sense of duty that makes them resistant to being sick or absent, which is routinely understood as a sign of weakness and further aggravates the vulnerability investigated here^(16)^. Alcoholic beverages and cigarettes are seen as an escape from these stressful routines as they provide relaxing and tranquilizing effects^(26)^. However, they are lifestyles that represent consistent risks for mental illness, which was found in this research, whose relationship does not predict illness, but can aggravate already installed psychophysical conditions, with nursing being highly susceptible to the consumption of these substances and with a tendency to suicide^(7,27–29)^.

This study showed that having a leisure routine is a protective factor for CMD and presenteeism, which corroborated with evidence that the non-existence of the practice is related to sleep problems and diagnoses of CMD, GAD and depression, in addition to the aforementioned practice being necessary for psychosocial well-being by minimizing stress symptoms^(17,23)^.

As limitations of this study, it is a convenience sample and the cross-section is highlighted, as it makes an in-depth analysis of cause and effect impossible. Cross-sectional studies using self-administered questionnaires may lead to inattention bias or misunderstanding in filling out. This important bias may be present even with the use of validated questionnaires and respecting the time for completion of each worker. The rate of refusals added to the questionnaires excluded due to incomplete completion totaled 36.4% of the population, which should also be considered as a limitation as it may impact the external validity of the findings. Another limiting factor was that the presence of workers with previously installed mental illness may have interfered in the analysis of the occurrence of CMD and loss of productivity.

The research demonstrated relevance for providing important scientific data about nursing workers’ mental health and its association with loss of productivity, work variables, health and presenteeism. CMD have become one of the main causes of productivity loss worldwide and yet there is a lack of research that seeks this relationship^(8)^. This theoretical consistency provides subsidies for planning and effective interventions that minimize the occurrence and progression of CMD and presenteeism in nursing workers, as well as helping to promote health and prevent consequential injuries, which lead to a significant loss of productivity, with a consequent increase in risks to workers’ health and the quality of care provided due to the greater occurrence of incidents and adverse events^(7,13)^.

## CONCLUSIONS

Presenteeism was a frequent phenomenon found in nursing workers and almost half of those affected carried out their activities with reduced work performance. The present study advances knowledge in the area of health and nursing by demonstrating that the occurrence of CMD in nursing workers was associated with presenteeism, with repercussions in a significant loss of productivity in the nursing team, impacts and limitations for tasks represented by the physical, mental and interpersonal domains, time management and production demand. Significant social and work factors that were also associated with the occurrence of mental disorders and presenteeism were represented by women, mothers, alcohol and tobacco professionals, with double employment and on night shifts (12/36).

The present study reinforces the urgent need for nursing work conditions related to the reduction of workload and the institution of a fair and adequate salary floor to be promptly instituted at the national level. It is also worth mentioning the diligence for the singular and individualized look at nurses and mothers, whose overload of motherhood combined with the precariousness of work may incur greater chances of presenteeism, occurrence of mental disorders and loss of productivity at work. Reviewing nursing workers’ policies and working conditions is essential so that quality care can be offered and disseminated. Future investigations are recommended in order to assess the impact of presenteeism and loss of productivity on patient safety and the quality of health care provided as well as nursing workers’ health.
